# Host Modulation by a Parasite: How *Leishmania infantum* Modifies the Intestinal Environment of *Lutzomyia longipalpis* to Favor Its Development

**DOI:** 10.1371/journal.pone.0111241

**Published:** 2014-11-03

**Authors:** Vania Cristina Santos, Vladimir Fazito Vale, Sydnei Magno Silva, Alexandre Alves Sousa Nascimento, Natalia Alvim Araujo Saab, Rodrigo Pedro Pinto Soares, Marilene Suzan Marques Michalick, Ricardo Nascimento Araujo, Marcos Horacio Pereira, Ricardo Toshio Fujiwara, Nelder Figueiredo Gontijo

**Affiliations:** 1 Department of Parasitology, Federal University of Minas Gerais (UFMG), Belo Horizonte, Minas Gerais, Brazil; 2 Institute of Biomedical Sciences, Federal University of Uberlandia (UFU), Uberlandia, Minas Gerais, Brazil; 3 Laboratory of Medical Entomology, Centro de Pesquisas Rene Rachou (CPqRR), Belo Horizonte, Minas Gerais, Brazil; 4 National Institute of Science and Technology-Molecular Entomology, Conselho Nacional de Desenvolvimento Cientifico e Tecnologico (CNPq), Brasilia, Distrito Federal, Brazil; Instituto Oswaldo Cruz, Fiocruz, Brazil

## Abstract

Some reports have described the interference of *Leishmania* on sand flies physiology, and such behavior most likely evolved to favor the development and transmission of the parasite. Most of these studies showed that *Leishmania* could modulate the level of proteases in the midgut after an infective blood meal, and decreased proteolytic activity is indeed beneficial for the development of promastigotes in the gut of sand flies. In the present study, we performed a detailed investigation of the intestinal pH in *Lutzomyia longipalpis* females naturally infected with *Leishmania infantum* and investigated the production of trypsin by these insects using different approaches. Our results allowed us to propose a mechanism by which these parasites interfere with the physiology of *L*. *longipalpis* to decrease the production of proteolytic enzymes. According to our hypothesis *L. infantum* promastigotes indirectly interfere with the production of trypsin by modulating the mechanism that controls the intestinal pH via the action of a yet non-identified substance released by promastigote forms inside the midgut. This substance is not an acid, whose action would be restrict on to release H^+^ to the medium, but is a substance that is able to interfere with midgut physiology through a mechanism involving pH control. According to our hypothesis, as the pH decreases, the proteolytic enzymes efficiency is also reduced, leading to a decline in the supply of amino acids to the enterocytes: this decline reduces the stimulus for protease production because it is regulated by the supply of amino acids, thus leading to a delay in digestion.

## Introduction

Sand flies from the genera *Phlebotomus* (Old World) and *Lutzomyia* (New World) serve as the vectors of many species of *Leishmania*. While feeding on an infected host, phlebotomine females ingest the amastigote form of the parasite, which, differentiates into the flagellated promastigotes in the sand fly gut lumen. Different forms of the promastigote appear during parasite development inside the gut lumen culminating with the appearance of the metacyclic promastigote, the form that infects the vertebrate host [1]–[3].

The host-parasite interaction is one of the most intimate ecological relationships between organisms. In this process, parasites develop mechanisms to better exploit their hosts, thereby increasing their chances of survival, whereas the hosts develop strategies against the parasite [4]. Many reports have described the effects of *Leishmania* infection on the physiology and behavior of sand flies. *Leishmania major* produces a substance capable of inhibiting the gut motility of *Phlebotomus papatasi* and other insect species [5]–[6], a phenomenon that would impair the elimination of promastigote forms during defecation. Additionally, *Leishmania* infection may increase the number of bites performed by the insect [7]–[8], thereby enhancing transmission.

In general, there is a consensus that sand fly infection could not be sustained if the *Leishmania* species were different from that naturally transmitted by the vector [9]. The first hypothesis to explain this specificity assumed that the digestive process of the vector would determine the development of the parasite [10]. The first experimental evidence for this phenomenon was obtained during the experimental infection of *P. papatasi* with *L. major* and *L. donovani* [11]: *L*. *major* is naturally transmitted by *P. papatasi*, though *L. donovani* is not able to develop in this sand fly species. The authors showed that *L. major* reduced the proteolytic activity of the insect's digestive enzymes, whereas *L. donovani* promoted an enhancement of the digestive activity. Furthermore, unidentified molecules (probably glycoconjugates) released into the culture medium by these two species of *Leishmania* reproduced this physiological effect when ingested by *P. papatasi* females. In another study [12], the use of Soybean Trypsin Inhibitor (SBTI) increased the survival of *L. donovani* in *P. papatasi*, suggesting that the modulation of proteases might somehow favor parasite development in a non-natural vector. The protective effect caused by unidentified molecules released in the intestine by promastigotes appears to be quite specific: the molecules released by a strain of *L. major* non-adapted to the *P. papatasi* population used in the experiments were not able to protect the parasite [13]. Similar results were obtained when *P. papatasi* and *Phlebotomus langeroni* were infected with *L. major*: the proteolytic activity was significantly lowered in *P. papatasi*, but remained the same in *P. langeroni*, which is not a natural vector of *L. major* [14]. In a study conducted a few years later, it became clear that sand flies fed a diet that disfavored the production of digestive proteases increased the survival of *L. donovani* in *P. papatasi* [15]. In contrast, diets that stimulated the production of proteases prevented the multiplication of the parasite in the vector.

Together, these data show that *Leishmania* is able to induce a decrease in the production of proteases during digestion and that this reduction is beneficial for the development of promastigotes in the gut of sand flies. Indeed, in the midgut, during differentiation from amastigotes to promastigotes, *L. major* parasites are very sensitive to the action of digestive proteases [16] and would therefore benefit from a decrease in proteolytic activity. Unlike these transition forms, fully differentiated amastigotes and promastigotes proved to be more resistant to the action of proteases but they could still suffer from their action [16]. A decline in the production of proteases would also lead to a delay in the digestive process and, consequently, the parasites would have more time to access the nutrients necessary for their development [14].

Studying the increased resistance of *L. major* promastigotes to proteases from *Phlebotomus duboscqi* (also a natural vector of *L. major*), Secundino and coworkers (2010) [17] observed that this process could be attributed to the production of membrane-bound proteophosphoglycans by the parasite. According the authors, the increased resistance did not occur via direct inhibition of trypsin by proteophosphoglycans or decreased production of the enzyme but through the coating of the parasites with these molecules. Again, the use of a protease inhibitor led to an increase in parasite numbers in the midgut [17].

*Lutzomyia longipalpis* is the main natural vector of *Leishmania infantum*, the etiological agent of American visceral leishmaniasis. A recent work in which *L. longipalpis* was infected with *L. mexicana* [18] or *L. infantum* [19] demonstrated the ability of these parasites to reduce the production of trypsin in the gut of infected insects. In the study with *L. mexicana*, the authors also showed that the number of parasites tends to increase when trypsin activity is lowered by RNAi [18].

Although it appears that *Leishmania* can modulate protease production, most likely through parasite-produced molecules, the mechanisms involved in this process are still unknown.

The intestinal pH of *L. longipalpis* is of great importance to the insect's digestive processes and probably can also influence the development of *Leishmania* in the gut [20]–[21]. In females fed with a non-blood diet, the thoracic and abdominal midguts are actively maintained at pH 6 by a so-called pH 6 maintenance mechanism [22]. However, this mechanism is switched off after a blood meal, and two mechanisms are then activated for the alkalinization of the abdominal midgut, thus affording normal digestion [22], [23]. The first alkalinization mechanism is not dependent on physiological changes in the intestine but on the volatilization of CO_2_ contained in the ingested blood, similar to the process of alkalosis when lungs are hyperventilated [22]. The second alkalinization mechanism results from a physiological alteration in the insect gut triggered by protein ingestion, a high concentration of amino acids also promotes lumen alkalinization [23].

In the present study, we performed a detailed investigation of the intestinal pH in *L. longipalpis* females naturally infected with *L. infantum* and also investigated the production of trypsin by these insects. The results allowed us to propose a mechanism by which *Leishmania* interferes with the sand fly physiology to decrease the production of proteolytic enzymes and thereby promote its development and transmission. According to our hypothesis, the promastigotes of *L. infantum* indirectly interfere with the production of trypsin by modulating the mechanism that controls the intestinal pH. This modulation occurs through the action of a substance released into the extracellular medium by the parasites. When the pH decreases, the activity of proteolytic enzymes is also reduced, consequently leading to a decrease in the supply of amino acids that reach the enterocytes. With fewer amino acids available, the stimulus for protease production also decreases, as it is regulated by the supply of amino acids to enterocytes. The decrease in the concentration of proteases promotes a delay in digestion, reducing the deleterious effects on the parasites. The presence of nutrients in the intestinal lumen for longer periods should favor promastigote development, as proposed by Dillon and Lane in 1993 [14].

## Results and Discussion

In blood-fed sand flies, all of the digested nutrients are absorbed and used by the insects to meet their metabolic needs and egg production. However, in *Leishmania*-infected sand fly females, it is assumed that there is competition for available nutrients between the host and the different forms of the parasite developing within the gut [14], [21]. Intestinal microorganisms such as commensal bacteria may also compete for the nutrients. Moreover, the different forms of the parasite may suffer from the deleterious action of digestive enzymes, particularly the amastigotes that are developing into promastigotes [16]. Within this context, parasite-driven changes in the intestinal environment that might favor them during development could have a major adaptive value.

In our experiments, we used three healthy dogs as controls and two symptomatic dogs with visceral leishmaniasis able to infect at least 85% of the *L. longipalpis* individuals fed on them. Among the infected female sand flies, 93.5% had more than 100 promastigotes in the intestinal lumen at five days after the meal. As noted by Dillon and Lane (1993) [14], the infected females in our experiments required significantly more time to complete blood digestion (Table I).

**Table I:** Delay in blood digestion in *Lutzomyia longipalpis* females infected with *Leishmania infantum*.

	Dissected midguts from blood-fed females observed between 73 and 76 hours after the blood meal	
Presence of blood under digestion	Uninfected	Infected
Yes	3	15
No	14	5
*Total*	*17*	*20*
Statistics	*p* = 0.0008*	

Fisher's exact test was used to compare the presence of digesting blood inside uninfected and infected females (*significant difference).

At least four different trypsin-like transcripts were identified in adult *L. longipalpis*' females in a transcriptome study (Lltryp1-Lltryp4) [24]–[25]. LlTryp2 transcripts are predominantly represented in sugar fed females while LlTryp1 was induced by blood ingestion and seems the principal isoform responsible for blood digestion [19], [25]. The other transcripts, LlTryp3 and LlTryp4 seems to be secondary isoforms and had a homogeneous sequence distribution between sugar and blood fed females [25].

**Figure 1:**
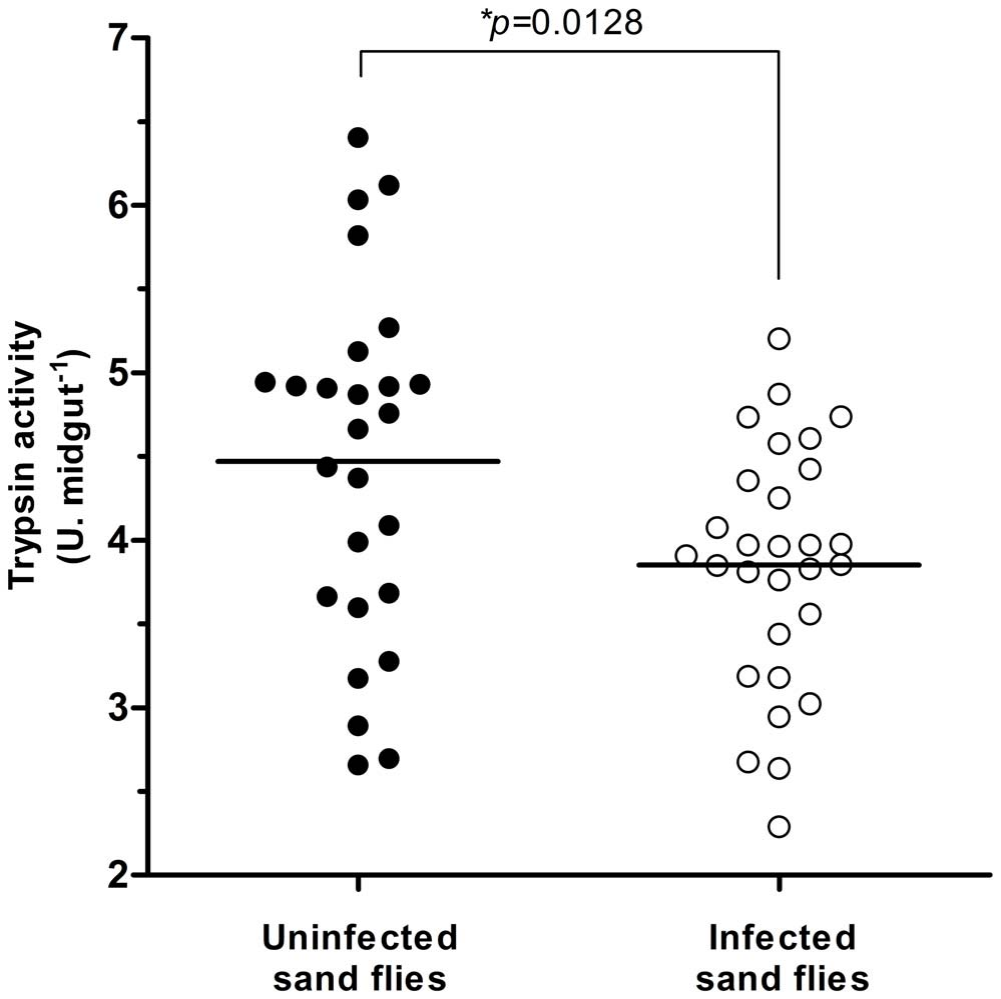
Evaluation of the trypsin activity in infected insects. Sand flies were dissected at 48 hours after feeding on an uninfected dog (n = 26) or a naturally infected dog (n = 29); the trypsin activity was measured using the synthetic substrate L-BApNA and was expressed as enzymatic units (U), normalized to the quantity of homogenate equivalent to one midgut (U.midgut^−1^). Each dot represents the activity obtained from an individual insect, and the horizontal line represents the mean value. The trypsin activity was significantly lower in the infected sand flies compared to the uninfected insects (*p* = 0.0128).

Trypsin-like enzymes are the major digestive proteases in sand flies, and their activity was compared between *Leishmania*-infected and uninfected females. It was evident that the infected females in our experiments produced less trypsin (Figure 1). We do not know which isoform of trypsin was decreased by the presence of *Leishmania*, but considering that LlTryp1 is induced by blood ingestion it is probably the isoform affected. A decrease in the proteolytic activity such that observed by us in the present study is similar to that observed by Schlein and Romano (1986) [11] in *P. papatasi* infected with *L. major* and more recently by Telleria and coworkers in 2010 [19] using *L. longipalpis* infected with *L. infantum* as a model. Different from previous results in which the insects were infected using artificial feeders, our data were obtained with sand flies infected by feeding on infected dogs. It is very likely that a decreased proteolytic activity in infected females (Figure 1) explains the observed increased digestion time (Table I), and a slight decrease in trypsin mRNA that was also observed in the midgut of *L. longipalpis* females infected with *L. infantum* during blood digestion [25]. Chymotrypsin activity was not evaluated in our experiments but its activity could have been also affected.

To investigate what occurs in the gut of *L. longipalpis* naturally infected with *L. infantum*, we evaluated another important physiological condition: the pH of the intestinal lumen during digestion. Although it was not possible to measure the pH throughout digestion in the present study, we observed that infected females have a significantly lower intestinal pH in comparison with the uninfected females between 48 and 55 hours after the blood meal (Table II). As shown previously by Santos and coworkers in 2008, *L. longipalpis* females have an intestinal pH of 6 prior to a blood meal [22]. In our experiments, after blood digestion, 100% of infected females analyzed showed an intestinal pH that was acidified to pH 6 at 5 to 6 days after bloodmeal (Table II). The same also occurred in uninfected insects (data not shown), in accordance with our previous prediction [21]. This re-acidification after digestion most likely plays an important role in the process of metacyclogenesis, as this process is stimulated by an acidification of the medium to pH ranging from 5.5 to 6.0 in cultured parasites [20], [26]–[27].

**Table II:** pH in the abdominal midgut of infected and uninfected *Lutzomyia longipalpis* females during and after blood digestion.

Physiological condition of the insects	pH measured in females fed in dogs		Statistics
	Uninfected	Infected	
During blood digestion (48–54 h after bloodmeal)	7.86±0.65 (n = 8)	6.84±0.28 (n = 10)	*p* = 0.0004*
After blood digestion (5–6 days after bloodmeal)	-	pH≤6 (n = 21)	-

Students' T test was used to compare the intestinal pH measured in uninfected and infected females (*significant difference).

In hematophagous insects, such as mosquitoes and other batch feeders, one of the factors controlling the production of digestive proteases is the supply of amino acids to the enterocytes [28]. *Aedes aegypti* females show no trace of trypsinolytic activity in the intestinal lumen prior to a blood meal [29]–[30]. In this insect, the production of digestive proteases occurs in two sequential steps [30]. First, the ingestion of blood with free amino acids promotes the translation of trypsin from mRNA molecules previously produced and stored in the enterocytes. In the second step, trypsin production is regulated transcriptionally and continues while there is a constant supply of amino acids to enterocytes [30]. If the amino acids supply to enterocytes is interrupted for any reason (e.g. by use of a high concentration of protease inhibitors) the interruption is interpreted as the end of digestion, and the mosquito defecates the blood without proper digestion [31].

**Figure 2:**
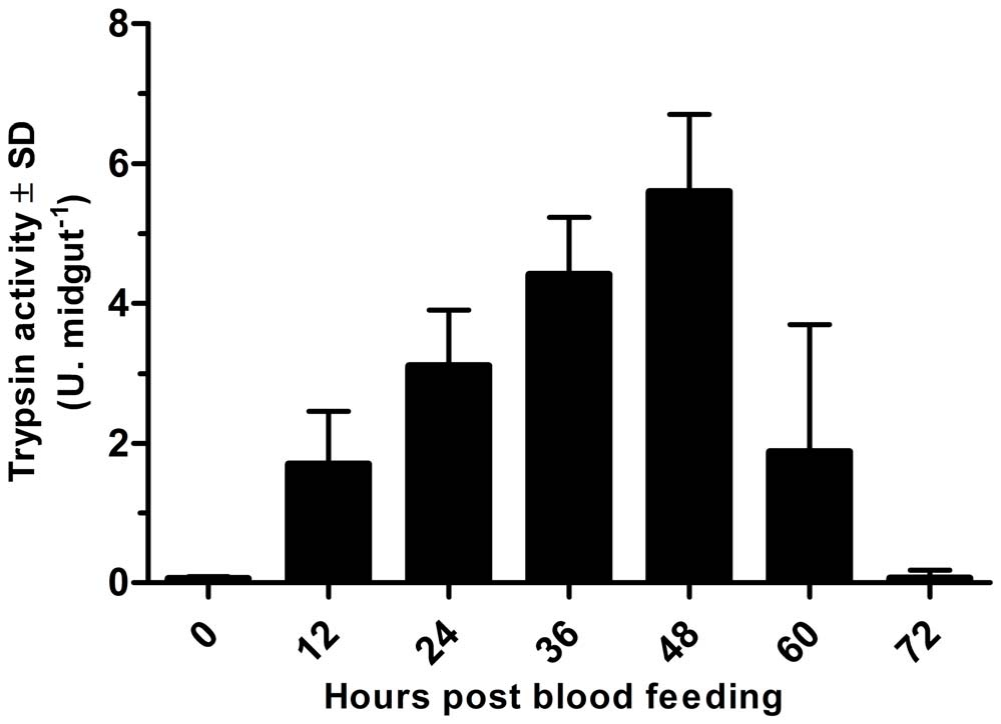
Trypsin activity during blood digestion in the *Lutzomyia longipalpis* midgut. Insects fed on an uninfected dog; 12 females were dissected every 12 h, and the trypsin activity was measured individually using the synthetic substrate L-BApNA. The activity is expressed as enzymatic units (U. midgut^−1^). The results are shown as the mean values ± standard deviation.

Our experiments showed that, unlike mosquitoes, unfed *L. longipalpis* females exhibited some trypsinolytic activity in the midgut (7.19+0.79 mU.midgut^−1^), which, which increased considerably after a blood meal from an uninfected dog (Figure 2).

**Figure 3:**
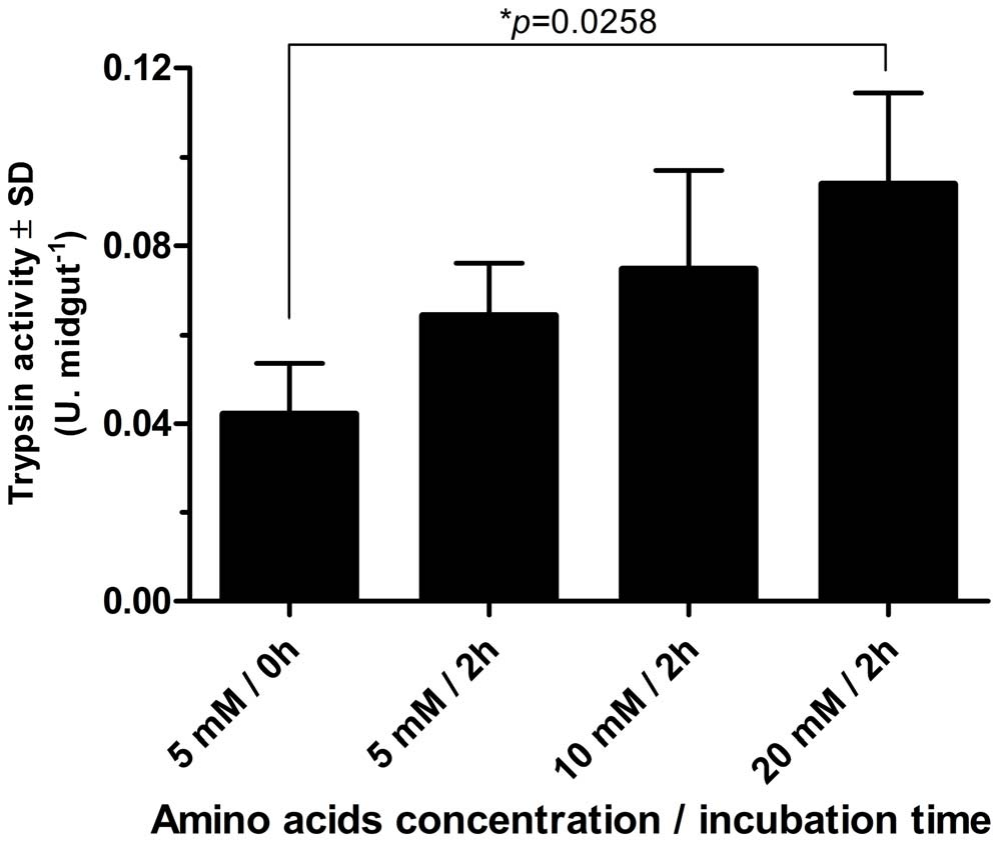
Evaluation of trypsin production induced by amino acids. *Lutzomyia longipalpis* midguts were dissected and incubated at 30°C for 2 hours in the presence of different concentrations of amino acids (5, 10, and 20 mM). The trypsin activity was measured using L-BapNA as the substrate and was expressed as enzymatic units (U), normalized to the quantity of homogenate equivalent to one midgut (U.midgut^−1^). The results are shown as the mean values of three independent experiments ± standard deviation. Trypsin activity from the midguts exposed to 20 mM amino acids was significantly different from the control (*p* = 0.0258).

The production of trypsin was found to be stimulated in *in vitro* preparations, whereby guts dissected from unfed females were incubated for 2 hours in M199 medium containing different concentrations of free amino acids. As trypsin production was proportional to the concentration of amino acids in the medium (Figure 3), it suggests that the supply of free amino acids to the enterocytes of *L. longipalpis* stimulates the production of trypsin by the midgut. One may speculate that the continuous supply of amino acids maintains the production of digestive proteases and that the intensity of the supply regulates the amount of enzyme produced. This hypothesis is consistent with the results presented in Figure 2, where trypsin production in female guts after feeding on an uninfected dog increases up to 48 hours. This peak in enzyme activity can be explained, at least in part, by the large supply of amino acids from the ongoing digestion. Endoproteases generate peptides that are digested to amino acids at the level of the intestinal microvilli by aminopeptidases and carboxypeptidases linked to the membranes of the enterocytes [14], [32]–[33]. These amino acids are then readily transported to the interior of enterocytes. However, with the decline in protein concentration during digestion, the amino acid concentration is also reduced, leading to a consequent decrease in protease production.

The kinase Target of Rapamycin (the TOR system), which is present in the midgut of insects, has been implicated as an amino acid sensor in different tissues [28]. It is possible that this signaling system is involved in the perception of increased concentrations of amino acids and even in the control of the intestinal pH in *L. longipalpis* and other insects

As in other insects, other factors may be involved in the induction of digestive proteases in *L. longipalpis* [34]–[36]. Although there are no reports in the literature, factors such as hormones produced by endocrine cells in the gut and other organs may play a complementary role that should be investigated in sand flies.

Considering that *L. infantum* causes gut acidification during digestion in infected females (Table II) we investigated whether this *Leishmania* species produces any substance capable of interfering with the mechanism of intestinal alkalinization that is triggered by a blood meal. In our experiments, the method used to simulate a blood meal consisted of forcing females to ingest a solution with a high concentration of amino acids using a forced-feeding apparatus [23]. The pH of the gut lumen was estimated by the color of the pH indicator dye Bromothymol Blue, which was dissolved in the amino acid-containing solution. The mechanism for pH 6 maintenance would be switched off when a female ingested the amino acid-containing solution, whereas the alkalinization mechanism would be turned on [23]. According to our results, the alkalinization process was partially impaired when females ingested a solution with *L. infantum* promastigote products (Table III). This phenomenon was not caused by an organic acid produced by the promastigotes because the pH of the solution was adjusted to 7.5 prior to use. Even with the pH adjusted to 7.5, a substance in the solution interfered with the alkalinization of the intestines preventing it to occur with the same efficiency observed in the control insects. This substance has a low molecular weight (equal or less than 3 KDa) and seems not to be peptidic in nature (Table III).

**Table III:** pH in the abdominal midgut of *Lutzomyia longipalpis* females fed on 100 mM amino acids solution plus 199 medium with and without *Leishmania* products submitted to different treatments.

	Dissected midguts from females observed immediately after ingestion of 100 mM amino acids solution mixed with:				
pH range	**A**: Insect Saline	**B**: 199 medium	**C**: 199 medium containing products released by *Leishmania*	**D**: 199 medium containing products released by *Leishmania* which were ultra-filtered	**E**: 199 medium containing products released by *Leishmania* which were digested by proteinase K and ultra-filtered
pH≤6.5	3	8	26	17	14
pH>6.5	13	27	12	4	11
*Total*	*16*	*35*	*38*	*21*	*25*
Statistics	**A/B** *p* = 1.00		**B/C** *p* = 0.0001*	**B/D** *p* = 0.0001*	**B/E** *p* = 0.014*

Obs.: Ingestion of 199 medium without amino acids did not alkalize midguts upper to pH 6.5 (data no show). Fisher's exact test was used to compare the treatments A B C D E (*significant difference).

It is important to note that, although the interference in the alkalinization process was only partial, it should have been sufficient to reduce the protease activity. Even a slight effect would be beneficial for the parasites because a very strong interference in pH could dramatically decrease proteolytic activity, causing the supply of amino acids to decline to a level that induces the defecation of the intestinal contents, as it occurs in *Aedes* females blood fed with a high concentration of protease inhibitors [31].

**Figure 4:**
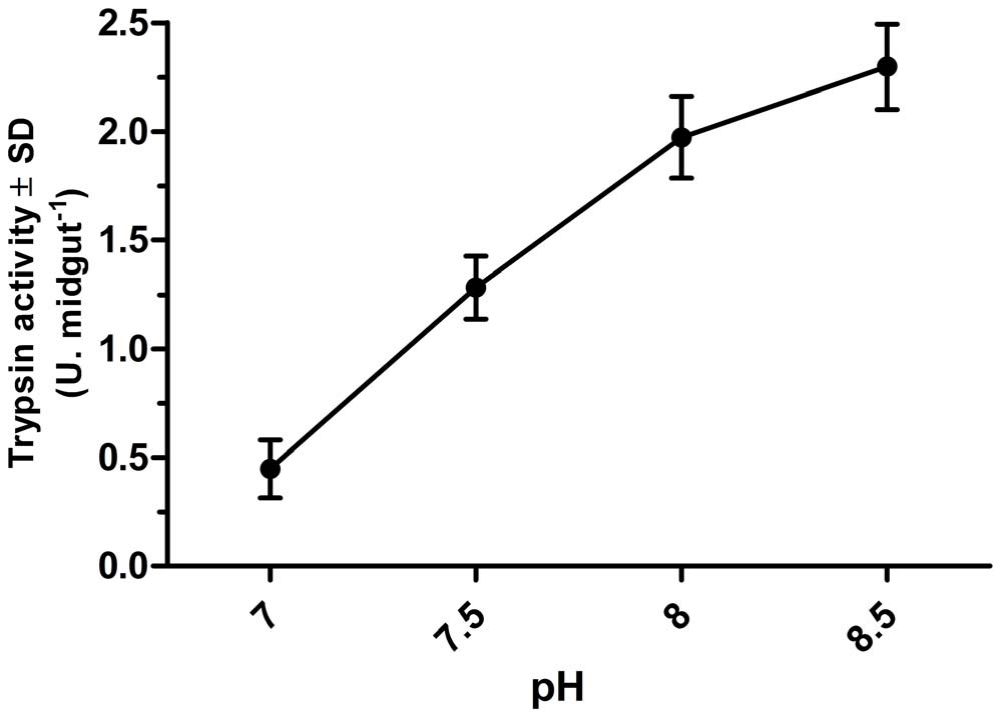
Activity of trypsin at different pH values. At 24 hours after a blood meal using hamsters, the midguts from 12 sand flies were dissected and pooled. The supernatant was incubated with L-BApNA at different pH values (7.0–8.5). The experiment was performed in duplicate and repeated independently three times. The graphic represents the mean values ± standard deviation.

Change in pH of an environment can dramatically influence enzyme activity, as it can be seen with trypsin and other insect digestive enzymes. The trypsinolytic activity in *L. longipalpis* is considerably higher at alkaline pH values (Figure 4). Thus, a decrease in intestinal pH would affect the digestion rate and consequently the production of amino acids that will be transported to the enterocytes. As shown in Figure 3, the supply of amino acids in *L. longipalpis* was important for inducing trypsin production. Therefore, the acidification of the gut promoted by *Leishmania* would reduce the production of free amino acids, which in turn would be responsible for the decreased trypsin production.

**Figure 5:**
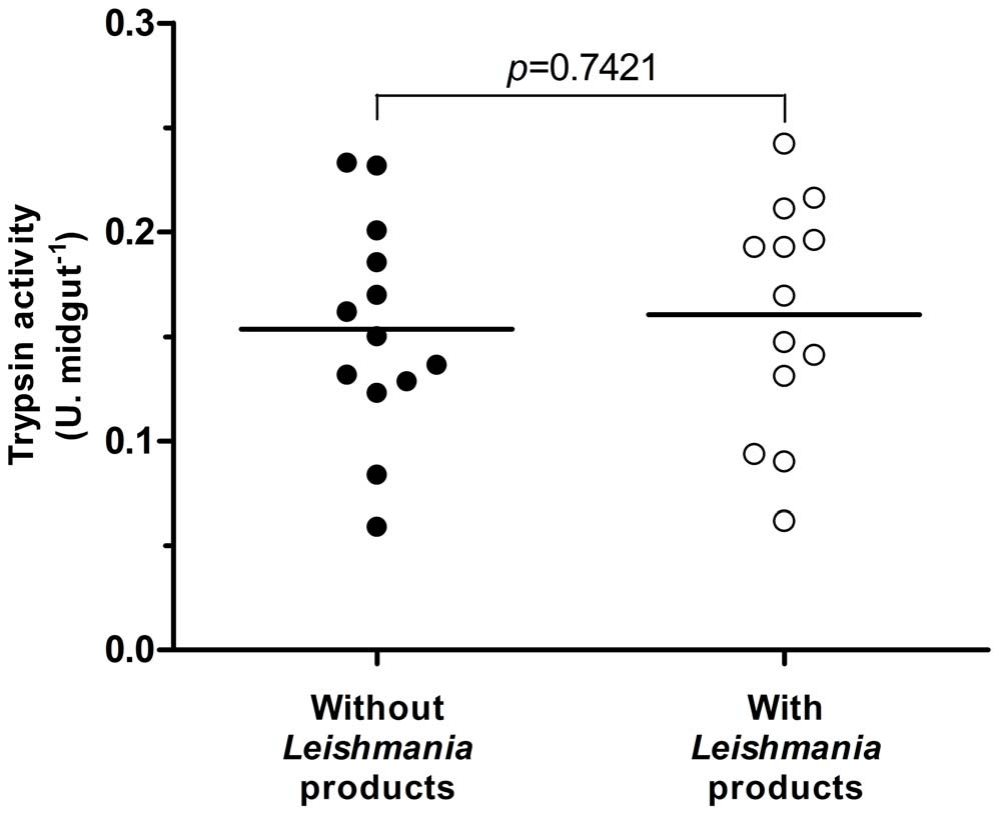
Influence of *Leishmania* products on midgut trypsin production. Midguts were exposed for 2 hours to *Leishmania infantum* products and to amino acids at a final concentration of 30 mM, and the trypsin activity was measured in the supernatant; no products from *Leishmania* were included the control group. Each dot represents the activity normalized to one midgut of 11 independent experiments. There was no difference in the quantity of trypsin produced (*p* = 0.7421).

Although our experiments show that *L. infantum* promastigotes may interfere with the host intestinal pH, decreased trypsin production could also be caused by the direct action of a substance secreted by the parasite on the mechanism of enzyme production by the enterocytes. Therefore, to clarify this issue, we performed amino acid-induced trypsin production experiments in the presence and absence of promastigote products. According to the results shown in Figure 5, there was no difference in the output of trypsin between the control group and the group containing the substances produced by *Leishmania*. This result indicates that the interference with trypsin production should be indirect and may occur due to a decrease in the pH value, as proposed above. In addition, *Leishmania* products cannot inhibit directly the trypsin activity. In enzymatic assays, using *L. longipalpis'* trypsin, the activity measured was 3.492 U.midgut^−1^±0.114 in the presence of *Leishmania* products and 3.547 U.midgut^−1^±0.126 in the absence (n = 15, *p* = 0.721).

The experiments by Schlein and Romano (1986) showed that *L. major* promastigotes produced a substance that interfered with the production of proteases in *P. papatasi*, its natural vector [11]. Thus, it is possible that a similar mechanism is operating in *L. longipalpis* infected with *L. infantum*.

During development within the gut of sand flies, *Leishmania* promastigotes produce several glycoconjugates. The best known is lipophosphoglycan (LPG), a molecule that is anchored in the parasite membrane and plays an important role during *Leishmania* development in the gut of its vectors [37]–[42]. According to Schlein and Romano (1986), the glycoconjugates produced by *L*. *major* appear to be molecules that interfere with the production of trypsin in *P. papatasi* [11]. We investigated whether purified LPG from *L. infantum* could interfere with the control of intestinal pH. If LPG is the molecule that interferes with pH physiology in the midgut, the alkalinization normally stimulated by amino acids would be impaired in comparison to the control group. Purified LPG was mixed with amino acids and offered through forced feeding to sand flies; however, there was no difference in the alkalinization process among the experimental groups (Table IV), leading to the conclusion that LPG is not the molecule that interferes with the alkalinization mechanism.

**Table IV:** pH in the abdominal midgut of *Lutzomyia longipalpis* females fed 199 medium plus 100 mM amino acids with or without lipophosphoglycan (LPG) purified from *Leishmania infantum*.

	Dissected midguts from females observed immediately after 199 medium ingestion	
pH range	Without LPG from *Leishmania*	With LPG from *Leishmania*
pH≤6.5	6	12
pH>6.5	18	14
*Total*	*24*	*26*
Statistics	*p* = 0.1485	

Fisher's exact test was used to compare the treatments.

## Conclusion

The results obtained in the experiments reported herein provide a good indication of the mechanism by which *L. infantum* promastigotes developing in the gut of *L. longipalpis* can control the production of the insect's digestive proteases to promote its own development and transmission. The promastigotes produce some as-yet-unidentified factor that can affect the mechanism of alkalinization in the vector intestine, thereby leading to reduced alkalinization. This poor alkalinization decreases the activity of proteases resulting in a decreased supply of amino acids to the enterocytes. Because the production of proteases is connected to the supply of amino acids, a reduced supply of amino acids promotes a decrease in the production of these enzymes, which favors the development of the parasites during digestion.

## Materials and Methods

### Insects and dogs

All experiments were performed with a *Lutzomyia longipalpis* colony originated from females collected in Teresina, state of Piauí, Brazil. Sand flies were maintained under controlled conditions according to Modi and Tesh (1983) [43]. The insects used were 3 to 5 days old at the beginning of the experiments and were fed according to the requirements of each assay.

Naturally infected dogs were previously selected to be our source of *Leishmania infantum* through a xenodiagnosis procedure according to Costa-Val and coworkers (2007) [44]. Two dogs able to infect up to 96% of the sand flies were chosen. Three other dogs free of infection were selected as controls.

The dogs used in the experiments were captured in the streets of Ribeirão das Neves, MG, Brazil by the city zoonosis control service or were given to the city zoonosis control service by their owners after a visceral leishmaniasis diagnostic, according to the current Brazilian laws. In this case, the owners were informed in detail about the possibility of the dogs be used for research purposes and signed a term of legal commitment. All procedures involving animals were approved by the Ethics Committee in Animal Experimentation at Universidade Federal de Minas Gerais (CETEA/UFMG) under study number 211/2007.

### pH measurement in the midguts of *Lutzomyia longipalpis* infected with *Leishmania infantum* during and after blood digestion

#### Sand fly infection

Dogs were subjected to general anesthesia using a mixture of 2 mg/kg of xylazine chlorhydrate and 11 mg/kg of ketamine chlorhydrate by intramuscular injection. The ears of anesthetized dogs were cleaned and shaved. Round plastic recipients (FleboContainers) containing approximately 30 females of *Lutzomyia longipalpis* were placed in direct contact with the internal surface of the ear skin and the sand flies were allowed to feed for 15 minutes on each ear. In the control group, sand flies fed on uninfected dogs [44].

#### pH measurement in sand fly abdominal midguts during blood digestion

Between 48 and 54 hours after the infective bloodmeal, the intestinal pH was measured in infected and uninfected females according to the methodology proposed by Santos and coworkers (2008) [22]. Briefly, a pair of microelectrodes (an H^+^ sensitive microelectrode and a reference microelectrode) previously calibrated were introduced in the blood bolus through the abdominal cuticle of sand flies. A voltmeter connected to the microelectrodes measured the voltage inside midgut in millivolts, and this voltage was transformed into pH units using a calibration curve. After the pH measurements, midgut contents from the infected group were examined under an optical microscope to confirm infection. Only data obtained from infected insects were considered.

#### pH measurement in sand fly midguts after blood digestion

Due to operational difficulties to measure the sand fly gut pH after blood digestion using microelectrodes, pH measurements were done using a pH indicator dye. Five days after the blood feeding, an unbuffered solution of 10% sucrose containing the pH indicator Bromothymol Blue (0.1%) with pH adjusted to approximately 7 was soaked in a piece of cotton and offered to the insects. After 24 hours, females were dissected under a stereomicroscope and the color of the dye inside the midgut was determined by comparison to buffered standard solutions prepared with the same dye at different pH values in 0.5-unit intervals [22]. After pH measurements, the midguts were examined under an optical microscope to confirm infection. Only the data obtained from infected insects were considered.

### Assays of trypsin activity (based on Fazito do Vale and coworkers (2007) [33])

#### Evaluation of the trypsin activity in infected insects

*Lutzomyia longipalpis* females were infected by feeding on naturally infected dogs, as above. At 48 hours after the infective bloodmeal, the females were dissected in 0.9% saline and washed in the same solution, and their midguts were individually transferred to 40 µL of 0.9% saline in microcentrifuge tubes. After homogenization by pipetting, 20 µL of each sample was collected and examined under an optical microscope to confirm the presence of *Leishmania* promastigotes. Only homogenates obtained from infected insects were used in the experiment. The remaining sample volume was re-adjusted to 125 µL with 0.9% saline and centrifuged at 14,000×*g* for 10 minutes at 4°C. The supernatant was collected and used in assays. The trypsin activity was assayed by mixing 100 µL of 0.1 M TRIS/HCl buffer (pH 7.5) (final concentration 50 mM), 40 µL of distilled water, 50 µL of sample (equivalent to 0.2 midgut), and 10 µL of 10 mM L-BApNA dissolved in DMSO (final concentration 0.5 mM) in a 96-well microplate. Blanks were prepared using 50 µL of 0.9% saline instead of the sample.

To investigate if trypsin inhibitors were produced by *Leishmania* during incubation, 20 µL of 199 medium containing *Leishmania* products were added to the assay mixture, above described, in the place of 20 µL of distilled water. In this case, trypsin was obtained from insects which were fed on hamsters. The control was prepared using 199 medium without *Leishmania* products.

The plate was incubated at 30°C for 30 minutes in a microplate reader (Molecular Devices/Versamax), and the absorbance was measured every 30 seconds at 415 nm. Trypsin activity was normalized and expressed as units per midgut (U.midgut^−1^). One unit of trypsin activity was defined as the amount of enzyme that produces 1 µmol of pNA per minute. Homogenates obtained from females that had fed on an uninfected dog were used as controls.

#### Trypsin activity during blood digestion

Sand flies were fed on an uninfected dog, and the trypsin activity was measured every 12 hours post-blood feeding. At each time point, ten insects were dissected, and their midguts were individually collected and transferred to 200 µL of 0.9% saline. After homogenization and centrifugation, 50 µL of the supernatant (equivalent to 0.25 of a midgut) was used in the assays, as described above. In these assays, 50 mM TRIS/HCl (pH 8.0) was used as the buffer. Trypsin activity was also measured in unfed females using a similar protocol.

#### Activity of trypsin at different pH values

At 24 hours after a blood meal using golden hamsters, dissected midguts of 12 females were pooled and homogenized in 2 mL of 0.9% saline. After centrifugation, 50 µL of the supernatant (equivalent to 0.5 of a midgut) was used in assays with 0.5 mM L-BApNA as the substrate (final concentration). Trypsin activity was evaluated using the same protocol described above, but different pH values were used (7.0, 7.5, 8.0, and 8.5, in 50 mM TRIS/HCl buffer, final concentration). Three independent experiments were performed.

#### Evaluation of trypsin production induced by amino acids

Three to 4 day old sugar-fed females were dissected in 0.9% saline, and their midguts were immediately transferred to microcentrifuge tubes containing 44 µL of 199 medium supplemented with glutamine (0.1 g/L) and antibiotics (5 µg of ampicillin and streptomycin); each tube contained 10 midguts. After dissection, the volume in the first tube was adjusted to 50 µL by the addition of more 6 µL of 199 medium; the final concentration of free amino acids in this tube was 5 mM (the concentration of free amino acids in 199 medium). In a second tube, the volume was completed by the addition of 4.5 µL of 199 medium and 1.5 µL of 172 mM stock solution of amino acids (Sigma; code M-5550); finally, the volume in a third tube was completed by the addition of 6 µL of 172 mM stock solution of amino acids. The final concentration of amino acids in each tube was 5, 10, and 20 mM, respectively. These tubes were then incubated at 28°C for 2 hours, and 100 µL of 0.1 M TRIS/HCl buffer (pH 8.5) containing 2% Triton X-100 was then added. The samples were sonicated for 10 seconds and centrifuged for 5 minutes at 12,000×*g* and 25°C. A 100 µL aliquot of the contents was transferred to a 96-well microplate, and 90 µL of 0.1 M TRIS/HCl buffer (pH 8.5) (without Triton X-100) was added; 10 µL of 0.25 M L-BApNA (dissolved in DMSO) was added to each well, and the plate was immediately transferred to a microplate reader. The plate was incubated at 30°C for 30 minutes, and the absorbance was measured at 415 nm every 30 seconds, with 10 s of automatic agitation just before each reading. Trypsin activity was normalized and expressed as units per midgut (U.midgut^−1^). An additional tube was prepared containing 5 mM amino acids but it was not incubated at 28°C during 2 hours as the other tubes.

### Evaluation of *Leishmania infantum* secreted products or purified lipophosphoglycan (LPG) on the mechanism for midgut pH control

To obtain secreted products, promastigotes of *Leishmania infantum* (MOHM/BR/1967/BH46) were initially grown in Grace's medium (Sigma G-9771) at pH 6.5 supplemented with 10% fetal bovine serum (FBS). Log phase parasites were washed by centrifugation at 2,000×*g* for 10 minutes at 20°C, and the promastigotes were resuspended in 10 mL of serum-free 199 medium (Sigma M-3769) supplemented with 100 U.mL^−1^ of penicillin G, and 100 µg.mL^−1^ of streptomycin. After a second centrifugation under the same conditions, the parasites were resuspended in 199 medium to a final concentration of 5×10^7^ promastigotes × mL^−1^. The mixture was transferred to a 20 mL culture flask and incubated for 24 hours at 24°C. After incubation, the medium was centrifuged (2,000×*g*, 10 min, 20°C), and the supernatant was aliquoted and maintained at −80°C until use.

To better characterize the molecule produced by *Leishmania*, part of the 199 medium containing *Leishmanias*'s products was ultra-filtered in an ultra-filter (Microcon Ultracel YM-3 3.000 MWCO Millipore) with a cutoff of 3 KDa or digested with proteinase K (0.4 U/mL) during 4 h at 37°C in pH 7.5 (10 mM HEPES Buffer) and ultra-filtered with a similar ultra-filter.

When phlebotomine sand flies ingest a solution of concentrated amino acids, their midguts undergo alkalinization [23], unless a substance in the medium interferes with this process. To investigate the potential to interfere with the mechanism of alkalinization, the supernatant of 199 medium containing *Leishmania* products or the same material treated as explained above was mixed with a 172 mM amino acid solution (Sigma M-5550) in a proportion of 1.0: 1.4 (the final concentration of amino acids was approximately 100 mM). The indicator dye Bromothymol Blue was added to a concentration of 0.1%. The pH was adjusted to 7.5 and the mixture containing *Leishmania* products was offered to 3 day-old females using the force-feeding technique described by Santos and coworkers (2008) [22]. Females in the control groups were fed with the same respective mixtures but without *Leishmania* products or with insect saline (119.7 mM NaCl, 2.68 mM KCl, 1.36 mM CaCl_2_ and 0.56 mM glucose). Immediately after feeding (each insect ingested approximately 0.5 µL), the females were dissected, and the pH inside the midgut was evaluated by the color of the dye [22]. The proportion of midguts in each pH category (pH<6.5 or pH≥6.5) was compared to the proportion observed in the respective control using Fisher's exact test.

The same procedure was used to evaluate the interference of lipophosphoglycan (LPG) from *L. infantum* (MHOM/BR/70/BH46) on the midgut pH. LPG was purified according to Coelho-Finamore and coworkers (2011) [42] and mixed with 199 medium containing amino acids (one volume of 199 medium and 1.4 volumes of amino acid stock solution; final concentration of 100 mM). The obtained solution (4 µg/mL LPG and 100 mM amino acids) was ingested by the insects using the forced feeding procedure [22].

In both experiments (assay with *Leishmania* products or LPG), control groups were prepared with the ingestion of 199 medium plus amino acids (final concentration 100 mM) without any other component or with insect saline.

### Evaluation of the ability of *Leishmania* products to directly influence trypsin production in the midgut

The products of *Leishmania infantum* in 199 medium were obtained as described above. Females (three to four days old) fed only with sugar were dissected, and their midguts (10 midguts) were opened, washed, and transferred to 35 µL of 199 medium supplemented with 0.1 g/L glutamine, 5.0 µg of ampicillin, and 5.0 µg of streptomycin. A 50-µL aliquot of 199 medium containing *Leishmania* products and 15 µL of amino acid stock solution were added to the mixture to a final amino acid concentration of 30 mM. After incubation at 28°C for 2 h, the midguts were mixed with 100 µL of 2% Triton X-100 in 0.1 M TRIS/HCl buffer (pH 8.5). The mixture was sonicated for 10 seconds and centrifuged for 5 minutes at 12,000×*g* at 25°C. A 100-µL aliquot of the supernatant containing the trypsin products from 5 midguts was collected, mixed with 90 µL of 0.1 M TRIS/HCl (pH 8.5), and 10 µL of 0.25 M L-BApNA dissolved in DMSO.

The plate was immediately incubated at 30°C for 30 minutes, and the absorbance was measured every 30 seconds at 415 nm, with 10 seconds of automatic agitation just before each reading. Activity was normalized and expressed as units per midgut (U.midgut^−1^). As a control, the same experiment was performed using medium 199 only instead of medium containing *Leishmania* products.

### Statistics

Comparisons among the averages were performed using Student's T-test, and differences were considered significant at *p*<0.05. Fisher's exact test was applied for the comparison of frequency distribution, with differences considered significant at *p*<0.05.
